# A Simple One-Pot Method for the Synthesis of BiFeO_3_/Bi_25_FeO_40_ Heterojunction for High-Performance Photocatalytic Degradation Applications

**DOI:** 10.3390/ijms26010196

**Published:** 2024-12-29

**Authors:** Yuan-Jun Song, Xiao-Ying Bi, Peng Xia, Fei Sun, Ze-Xian Chen, Xiao-Yang Zhang, Tong Zhang

**Affiliations:** 1 Joint International Research Laboratory of Information Display and Visualization, School of Electronic Science and Engineering, Southeast University, Nanjing 210096, China; songyuanjunwf@163.com (Y.-J.S.); 230238445@seu.edu.cn (X.-Y.B.); 220221657@seu.edu.cn (P.X.); sunfei202302@163.com (F.S.); zed.chen@foxmail.com (Z.-X.C.); zxycom@seu.edu.cn (X.-Y.Z.); 2 Suzhou Key Laboratory of Metal Nano-Optoelectronic Technology, Suzhou Campus, Southeast University, Suzhou 215123, China; 3 Key Laboratory of Micro-Inertial Instrument and Advanced Navigation Technology, Ministry of Education, School of Instrument Science and Engineering, Southeast University, Nanjing 210096, China

**Keywords:** Bi-based metal oxide, heterojunction, photocatalysis, degradation of organics

## Abstract

This study presents a facile one-pot synthesis method to fabricate BiFeO_3_-Bi_25_FeO_40_-Bi_2_O_3_ heterojunction photocatalysts with controllable compositions and pure phases. Three different binary heterojunctions (BiFeO_3_/Bi_25_FeO_40_, BiFeO_3_/Bi_2_O_3_, and Bi_25_FeO_40_/Bi_2_O_3_) and a ternary BiFeO_3_/Bi_25_FeO_40_/Bi_2_O_3_ heterojunction were formed, all exhibiting significantly enhanced photocatalytic performance for the degradation of methylene blue (MB) and phenol under visible light irradiation, outperforming the individual compositions. Notably, the BiFeO_3_/Bi_25_FeO_40_ heterojunction achieved the highest degradation efficiency (93.68% and 83.3% for MB and phenol, respectively) as well as excellent stability. Impressively, the phenol degradation efficiency of BiFeO_3_/Bi_25_FeO_40_ was even over twice that of BiFeO_3_ and Bi_25_FeO_40_, and four times higher than that of Bi_2_O_3_. The enhanced photocatalytic activity of the BiFeO_3_/Bi_25_FeO_40_ heterojunction is primarily attributed to the advantageous S-scheme band alignments that facilitate efficient charge separation and enhance redox capabilities. While other heterojunctions also exhibited improved MB and phenol degradation efficiency, each unique combination of materials led to distinct electronic structures and diverse reaction mechanisms. The simplicity and scalability of the synthesis method, combined with the remarkable photocatalytic performance of these BiFeO_3_-Bi_25_FeO_40_-Bi_2_O_3_ heterojunction materials, position them as highly promising candidates for applications in environmental remediation and solar energy conversion.

## 1. Introduction

Developing efficient and stable photocatalysts for the degradation of organic pollutants in water has been a significant area of research in recent decades [1,2,3]. Semiconductor photocatalysts hold significant potential as they can harness solar energy to facilitate redox reactions, efficiently breaking down various organic pollutes such as dyes, pesticides, and pharmaceuticals [4,5]. Titanium dioxide (TiO_2_) has long been recognized as an n-type semiconductor with remarkable photocatalytic properties, sparking extensive research since the 1970s and presenting significant potential across various domains, including environmental protection and energy conversion [6,7,8]. However, despite the sensitivity of TiO_2_ to sunlight, its wide bandgap of 3.2 eV restricts its absorption primarily to the ultraviolet spectrum. This limitation significantly curtails its applicability within the visible light range, presenting numerous challenges in practical settings. Furthermore, the pronounced recombination of electrons and holes contributes to relatively subdued photocatalytic efficiency, adversely affecting its overall performance in photocatalytic reactions. These factors have long impeded the widespread deployment of TiO_2_ in applied contexts. In a concerted effort to rectify these shortcomings, researchers such as Wang et al. have synthesized a variety of modified TiO_2_ nanotubes, including Bi_2_S_3_-BiOBr/TiO_2_ NTA and TiO_2_ NTs/Sn_3_O_4_, which have shown significant enhancements in light absorption capabilities and electron transport properties of TiO_2_ under visible light exposure. These modified TiO_2_ nanotubes have exhibited exceptional efficacy in the photocatalytic degradation of organic pollutants, such as phenol and methylene blue (MB), thereby underscoring their promising prospects for real-world applications. Nevertheless, the preparation processes for these modified materials are often intricate, typically involving multi-step synthesis, which escalates production costs and demands considerable time investment. Moreover, during the photocatalytic process, these modified materials frequently necessitate the application of an external voltage and electrolytes to sustain their reactivity, thus partially limiting their practical feasibility for large-scale implementation [9,10]. Recently, Bi-based oxides have garnered considerable attention as potential promising photocatalysts due to their straightforward synthesis processes, cost-effectiveness, unique electronic properties, and excellent visible light response, coupled with their remarkable catalytic characteristics [11,12,13,14].

Among these materials, Bi_2_O_3_ is notable due to its remarkable oxygen storage capacity and beneficial redox properties. However, its wide band gap of approximately 2.7 eV restricts its potential in photocatalytic applications [12,15]. Consequently, research has shifted towards bismuth-based metal oxides such as BiFeO_3_ and Bi_25_FeO_40_, which promise improvements in bandgap characteristics and light response efficiency. BiFeO_3_, a multiferroic material with a perovskite structure, shows strong light absorption across the visible spectrum [16,17]. This unique structure not only provides catalytically active sites but also benefits from intrinsic ferroelectric and magnetic properties [17,18]. These features help BiFeO_3_ separate and transfer charges effectively, making it a great candidate for photocatalytic uses. Recent studies have demonstrated its effectiveness in degrading organic pollutants under visible light irradiation, showcasing its potential for environmental remediation [19]. However, challenges regarding its stability and efficiency under prolonged light exposure, as well as its tendency for phase separation at elevated temperatures, must be addressed to enhance its practical applications [20,21]. Similarly, Bi_25_FeO_40_ has emerged as an important material in the field of photocatalysis because of its complex crystal structure, which may offer synergistic effects between Bi and Fe [22,23,24,25,26,27,28]. The sillenite-type structure of Bi_25_FeO_40_ provides active surface reaction sites for the adsorption and activation of organic pollutants. Additionally, the presence of Fe enhances its magnetic properties, which could also lead to improved charge transport and reduced recombination rates of electron–hole pairs. However, despite the individual merits of BiFeO_3_ and Bi_25_FeO_40_, the photocatalytic performance of these individual Bi-based oxides is often limited by factors such as rapid recombination of photogenerated electron–hole pairs and suboptimal light harvesting.

To address these limitations, people developed heterojunction structures composed of Bi-based materials, which show enhanced photocatalytic efficiency. For example, many studies have demonstrated the potential for photocatalytic degradation of organic pollutes by modifying Bi_2_O_3_ materials by incorporation with another composition. For instance, a CaBi_6_O_10_/Bi_2_O_3_ heterojunction prepared by the simple deposition method exhibited better photocatalytic properties for MB degradation in water under visible light due to effective electron–hole separation compared to single-phase ones [29]. Additionally, heterostructures such as Bi_2_O_3_/BiOCl [30], Bi_2_O_3_/Bi_2_SiO_5_ [31], and Bi_2_O_3_/PbS [10] have been reported to exhibit enhanced charge separation and transfer efficiency, improved photocatalytic activity, and superior photoelectrochemical performance. Similarly, several studies have highlighted the advantages of integrating Bi_25_FeO_40_ with other materials to further boost its photocatalytic effectiveness. For example, Bi_25_FeO_40_/graphene and Bi_25_FeO_40_/Cu_2_O have exhibited enhanced photocatalytic effects, largely attributed to the more efficient separation of photogenerated electrons and holes than Bi_25_FeO_40_ [24,25]. This suppression of electron–hole recombination leads to a higher concentration of photogenerated carriers and improved photocatalytic efficiency. Moreover, Shi et al. synthesized a BiFeO_3_/Bi_2_O_3_ heterojunction with high photocatalytic activity and stability using the sol–gel technique. This heterojunction exhibits excellent visible light absorption, which improves its photocatalytic performance [32]. Wang et al. also employed the sol–gel method to precisely tune the bandgap of the BiFeO_3_/Bi_25_FeO_40_ heterojunction by adjusting the concentration of Fe^3+^ and the calcination temperature [25]. Despite these efforts, comprehensive studies on the synergistic effects of composition and heterojunction structure on photocatalytic properties are rarely reported in the literature. This is because the synthesis of binary or ternary BiFeO_3_-Bi_25_FeO_40_-Bi_2_O_3_ heterojunctions with controllable compositions presents a challenge due to the propensity for phase transformation within the Bi-Fe-O system [33].

In this study, we present a straightforward method for synthesizing BiFeO_3_-Bi_25_FeO_40_-Bi_2_O_3_ heterojunction photocatalysts, allowing for precise control over their composition. We systematically investigated how variations in the composition of heterojunctions influence photocatalytic performance, aiming to identify the most promising materials for photocatalytic degradation. MB and phenol were selected as probe molecules. It is important to note that while MB is commonly used as a probe in photocatalytic degradation studies, it can also undergo degradation through a photosensitization pathway, which may impact the accuracy of our conclusions. Therefore, we also evaluated the photocatalytic activity of the as-prepared catalysts using colorless phenol, as it has a negligible photosensitizing effect under visible light. A comprehensive understanding of the reaction mechanisms associated with different types of heterojunctions in the degradation of organic was explored through extensive characterizations of the materials, including their morphology, crystal structure, electronic features, optical properties, and electrochemical behavior. These findings will enhance our understanding of the design principles for heterojunction photocatalysts and their potential applications in environmental remediation and renewable energy technologies, thus paving the way for future advancements in sustainable materials science.

## 2. Results and Discussion

**Figure 1:**
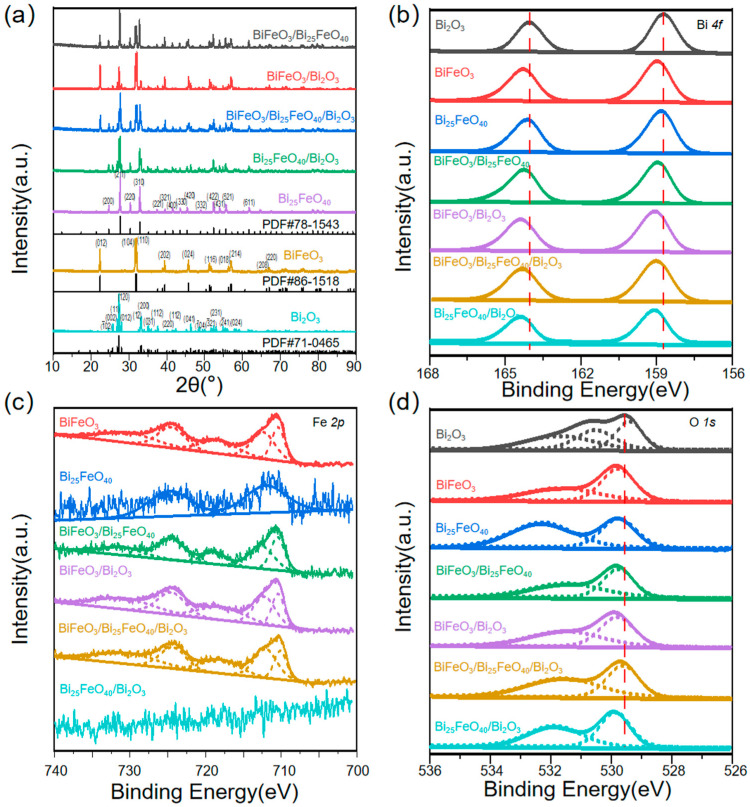
(**a**) XRD patterns and XPS spectra of (**b**) Bi 4f, (**c**) Fe 2p, and (**d**) O 1s for Bi_2_O_3_, BiFeO_3_, Bi_25_FeO_40_, BiFeO_3_/Bi_25_FeO_40_, BiFeO_3_/Bi_2_O_3_, Bi_25_FeO_40_/Bi_2_O_3_, and BiFeO_3_/Bi_25_FeO_40_/Bi_2_O_3_.

The XRD results in Figure 1a provide compelling evidence for the successful formation of pure phases of BiFeO_3_ and Bi_25_FeO_40_, as well as the presence of various heterojunction structures. For pure BiFeO_3_ and Bi_25_FeO_40_, all characteristic diffraction peaks can be perfectly indexed to the rhombohedral structure of BiFeO_3_ (JCPDS card No. 86-1518) and the body-centered cubic structure of Bi_25_FeO_40_ (JCPDS card No. 78-1543), respectively. Four heterojunctions of BiFeO_3_/Bi_25_FeO_40_, BiFeO_3_/Bi_2_O_3_, Bi_25_FeO_40_/Bi_2_O_3_, and BiFeO_3_/Bi_25_FeO_40_/Bi_2_O_3_ were achieved with nominal loading after calcination at different temperatures. BiFeO_3_/Bi_2_O_3_ was produced at 600 °C with Bi:Fe = 1:1 molar ratio loading. When the temperature was increased to 700 °C, the partial BiFeO_3_ phase transferred to the Bi_25_FeO_40_ phase, producing the BiFeO_3_/Bi_25_FeO_40_/Bi_2_O_3_ heterojunction. When the temperature increased further to 750 °C, the Bi_2_O_3_ was completely decomposed, leading to the formation of BiFeO_3_/Bi_25_FeO_40_. In contrast, Bi_25_FeO_40_/Bi_2_O_3_ was produced at 600 °C with Bi:Fe = 25:1 loading, and the phase could be maintained with an increased calcination temperature at 700 °C. This is because, according to the phase diagram of Bi-Fe-O compounds, BiFeO_3_ is the metastable phase, which means it can be readily decomposed to other phases such as Bi_25_FeO_40_ without the precise control of synthesis conditions [33]. The final atomic ratios of Bi:Fe in BiFeO_3_/Bi_25_FeO_40_, BiFeO_3_/Bi_2_O_3_, and BiFeO_3_/Bi_25_FeO_40_/Bi_2_O_3_ heterojunctions were 2.3:1, and the ratio in Bi_25_FeO_40_/Bi_2_O_3_ was 50.4:1. The sharp and well-defined peaks observed in the XRD patterns indicate high crystallinity and phase purity. Furthermore, the transition between these phases suggests a well-controlled synthesis process conducive to the formation of heterojunctions, which are critical for enhancing optical and electrical properties.

XPS analysis was conducted to evaluate the element oxidation state and binding energies of materials. The XPS spectra for Bi 4f, shown in Figure 1b, display two distinct peaks: Bi(4f_5/2_) at 164.45–164.05 eV and Bi(4f_7/2_) at 159.10–158.73 eV. These data confirm the existence of Bi^3^⁺ ions in Bi_2_O_3_, BiFeO_3_, and Bi_25_FeO_40_ [34]. In the case of the Fe 2p XPS spectra for BiFeO_3_, Bi_25_FeO_40_, and the BiFeO_3_/Bi_25_FeO_40_/Bi_2_O_3_ systems (see Figure 1c), multiple signals were detected. Notably, the Fe 2p_3/2_ binding energy for BiFeO_3_ is resolved into two significant peaks: one at 724.51 eV, which corresponds to Fe^2^⁺ 2p_1/2_, and another at 710.70 eV associated with Fe^3^⁺ 2p_3/2_ [25,35]. Additionally, these peaks can also be observed at the BiFeO_3_–Bi_25_FeO_40_–Bi_2_O_3_ heterojunctions. The signal for Fe 2p_3/2_ in the Bi_25_FeO_40_ sample is notably weaker due to lower atomic concentration, a trend also seen in Bi_25_FeO_40_/Bi_2_O_3_, although it is still detectable. The O 1s XPS spectra, illustrated in Figure 1d, feature a dominant peak at 529.93–529.53 eV, which is attributed to lattice oxygen. Meanwhile, additional peaks at 532.03–531.70 eV suggest the presence of chemisorbed oxygen connected to oxygen vacancies [25,36]. BiFeO_3_/Bi_25_FeO_40_ exhibits slight shifts in binding energies compared to BiFeO_3_ and Bi_25_FeO_40_, likely due to charge transfer interactions between these Bi-Fe oxides. This observation suggests enhanced electronic coupling within the heterojunctions, which may improve their photocatalytic efficiency and overall performance. In contrast, the binding energy shifts in BiFeO_3_/Bi_2_O_3_ and Bi_25_FeO_40_/Bi_2_O_3_ are more pronounced. This is attributed to the weaker interaction between BiFeO_3_ or Bi_25_FeO_40_ and Bi_2_O_3_, potentially leading to reduced tunability of optical and electronic properties, as well as stability.

**Figure 2:**
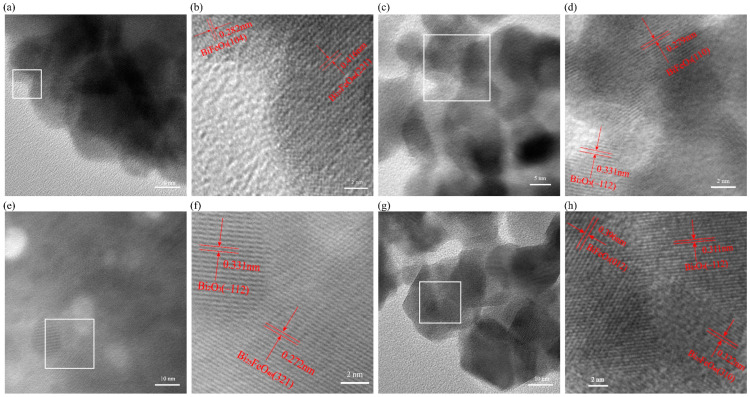
TEM images at low and high magnifications of (**a**,**b**) BiFeO_3_/Bi_25_FeO_40_; (**c**,**d**) BiFeO_3_/Bi_2_O_3_; (**e**,**f**) Bi_25_FeO_40_/Bi_2_O_3_; and (**g**,**h**) BiFeO_3_/Bi_25_FeO_40_/Bi_2_O_3_.

The composition of the BiFeO_3_-Bi_25_FeO_40_-Bi_2_O_3_ system heterojunctions was also verified through TEM characterization. This analysis reveals distinct morphologies and the grinding of crystal lattices, highlighting the interfaces between the various phases. For instance, Figure 2a clearly illustrates the heterojunction structure of BiFeO_3_/Bi_25_FeO_40_, comprising BiFeO_3_ (104) and Bi_25_FeO_40_ (221) orientations. Likewise, Figure 2b–d provide clear delineations of other heterojunctions, including BiFeO_3_ (110)/Bi_2_O_3_ (−112), Bi_25_FeO_40_ (321)/Bi_2_O_3_ (−112), and BiFeO_3_ (012)/Bi_25_FeO_40_ (310)/Bi_2_O_3_ (−112). These observations are critical for understanding the structural characteristics of the heterojunctions within the system. Moreover, the morphologies of all materials are sheet-like with a size in a range of 250–600 nm, which is beneficial for enhancing photocatalytic activity. The high surface area-to-volume ratio of the nanosheets provides more active sites for the adsorption and degradation of pollutants. Additionally, the thin sheet-like structure facilitates the rapid transport of photogenerated charge carriers to the surface, minimizing recombination losses and improving the overall efficiency. This unique morphology, combined with the synergistic effects of the BiFeO_3_-Bi_25_FeO_40_-Bi_2_O_3_ heterojunction, represents a powerful strategy for developing high-performance photocatalysts.

## 3. Photocatalytic Performance

**Figure 3:**
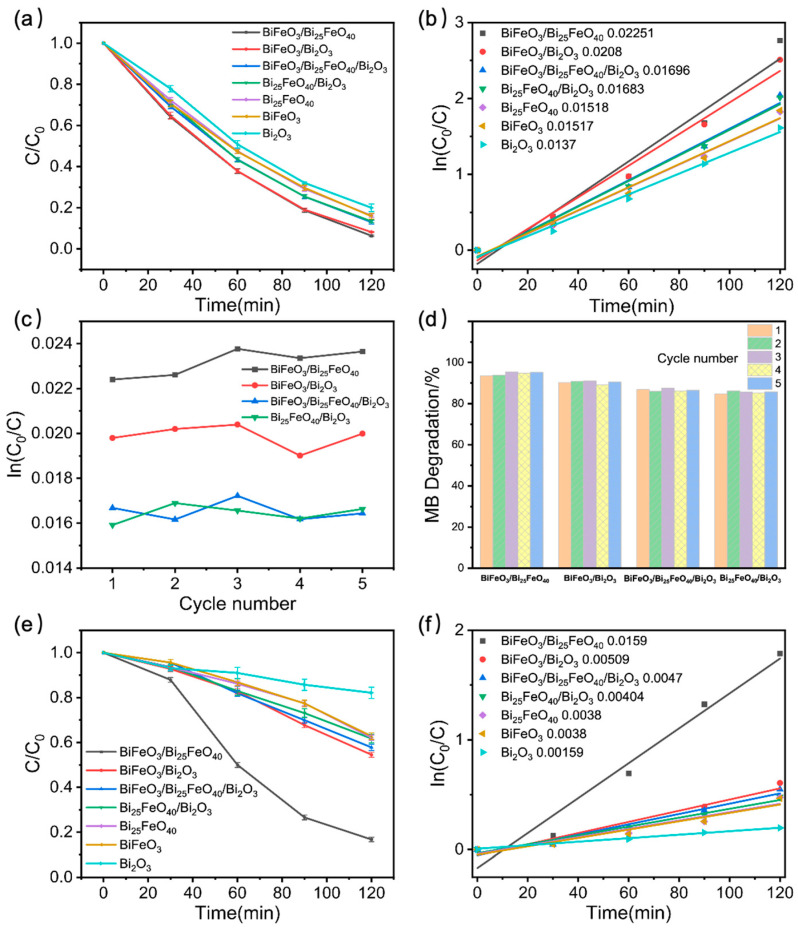
(**a**) The concentration changes of MB (C/C_0_); (**b**) the rate constant ln(C_0_/C) curves as a function of time; (**c**,**d**) recycle test over four heterojunctions repeated 5 times in the MB degradation; (**e**) the concentration changes of phenol (C/C_0_) and (**f**) the rate constant ln(C_0_/C) curves as a function of time in the phenol degradation over Bi_2_O_3_, BiFeO_3_, Bi_25_FeO_40_, BiFeO_3_/Bi_25_FeO_40_, BiFeO_3_/Bi_2_O_3_, Bi_25_FeO_40_/Bi_2_O_3_, and BiFeO_3_/Bi_25_FeO_40_/Bi_2_O_3_.

The photocatalytic performance of the synthesized BiFeO_3_-Bi_25_FeO_40_-Bi_2_O_3_ heterojunction system was evaluated through the degradation of MB and phenol under visible light irradiation for 120 min. Figure 3a illustrates the boosted enhancements in photocatalytic activity of prepared heterojunction samples compared to pure BiFeO_3_, Bi_25_FeO_40_, and Bi_2_O_3_ benchmarks. The photocatalytic degradation rates of MB over BiFeO_3_/Bi_25_FeO_40_, BiFeO_3_/Bi_2_O_3_, BiFeO_3_/Bi_25_FeO_40_/Bi_2_O_3_, and Bi_25_FeO_40_/Bi_2_O_3_ heterojunctions were 93.68%, 91.86%, 87.03%, and 86.63%, respectively. On the other hand, the degradation rates of BiFeO_3_, Bi_25_FeO_40_, and Bi_2_O_3_ were 83.85%, 84.16%, and 80.05%, respectively (see Figure S1j). The results indicate that among the materials tested, BiFeO_3_/Bi_25_FeO_40_ demonstrates the highest performance with an impressive degradation efficiency. The rate constants of degradation over these catalysts were estimated by fitting using the pseudo-first-order reaction kinetic model (see Figure 3b), ln(C_0_/C) = *k*t, where *k* is the reaction rate constant in the unit of min^−1^, *C*_0_ is the initial concentration, and *C* is the concentration at time *t*. The rate constant of MB degradation of BiFeO_3_/Bi_25_FeO_40_ (0.02251 min^−1^) was much higher than that of Bi_2_O_3_ (0.0137 min^−1^). This significant enhancement can largely be attributed to improved charge separation and a greater absorption of visible light. On the one hand, the heterojunction interface can facilitate the transfer of photogenerated charge carriers, thereby reducing recombination losses and increasing the availability of reactive species essential for degradation. On the other hand, the combination of BiFeO_3_ and Bi_25_FeO_40_ may lead to a broader light absorption spectrum, enabling the composite to utilize a wider range of the solar spectrum. Additionally, the optimal lattice matching between BiFeO_3_ and Bi_25_FeO_40_ may create synergistic effects that further enhance photocatalytic activity. These effects could include improved charge transport, increased density of active sites, and better stability during the degradation process. The BiFeO_3_/Bi_2_O_3_ heterojunction also exhibited a similar structure and intrinsic features, resulting in an increased degradation rate of 0.0208 min⁻^1^. This phenomenon has also been encountered in other reported Bi_2_O_3_-based heterojunction photocatalysts, such as BiVO_4_/Bi_2_O_3_ and WO_3_/Bi_2_O_3_. BiVO_4_/Bi_2_O_3_ heterojunctions synthesized by the hydrothermal method show enhanced photocatalytic activity in research, mainly due to the effective separation of photogenerated carriers, as found in studies under different synthesis strategies [37,38]. Furthermore, Bi_2_O_3_/WO_3_ heterojunctions synthesized by the hydrothermal method or be constructing porous microspheres exhibit better performance in photocatalytic research because of the effective separation of photogenerated carriers [39,40].

In contrast, the photocatalytic activities of BiFeO_3_/Bi_25_FeO_40_/Bi_2_O_3_ and Bi_25_FeO_40_/Bi_2_O_3_, measured at 0.01696 and 0.01683 min^−1^, respectively, exhibited slight changes compared to their individual constituent compositions. This is likely a result of the crystal mismatch between the different phases or the development of an unsuitable new electronic structure within the composite materials. In addition, these observed discrepancies in performance between these heterojunctions indicate a significant influence of compositional variations on their absorption efficiency of solar energy and effective charge transport. Therefore, it is important to optimize the composition of photocatalysts to enhance their photocatalytic efficiency. Furthermore, as shown in Figure 3c,d, we also evaluated the cycling performance of four distinct heterojunction photocatalysts. After five cycles of photodegradation, the high MB degradation rate remained, indicating that these photocatalysts possess good stability.

To assess the photocatalytic activity of these catalysts without the influence of photosensitization, the degradation of phenol was also investigated. The degradation trends observed in relation to the composition of the heterojunctions were consistent with those during the MB experiments (see Figure 3e,f). Significantly, the degradation rate of phenol using BiFeO_3_/Bi_25_FeO_40_ reached 83.3%, which was 2.2 times greater than that of BiFeO_3_ (37.9%) and Bi_25_FeO_40_ (37.9%), and 4.6 times higher than that of Bi_2_O_3_ (17.9%). This finding further substantiates the superior photocatalytic activity of BiFeO_3_/Bi_25_FeO_40_. The degradation rates for the other heterojunctions, BiFeO_3_/Bi_2_O_3_, BiFeO_3_/Bi_25_FeO_40_/Bi_2_O_3_, and Bi_25_FeO_40_/Bi_2_O_3_, were 45.5%, 42.2%, and 38.2%, respectively, indicating notable improvements as well. Additionally, we summarized the photocatalytic performance of MB and phenol degradation (including degradation rate, efficiency, light source, and synthesis method) over various catalysts reported in the literature in Table 1, comparing these findings with our results. To enhance clarity, the degradation rate was normalized to min⁻^1^·g⁻^1^. It is evident that the photocatalysts developed in this study, particularly BiFeO_3_/Bi_25_FeO_40_, exhibit better performance than those in most recent reports.

**Figure 4:**
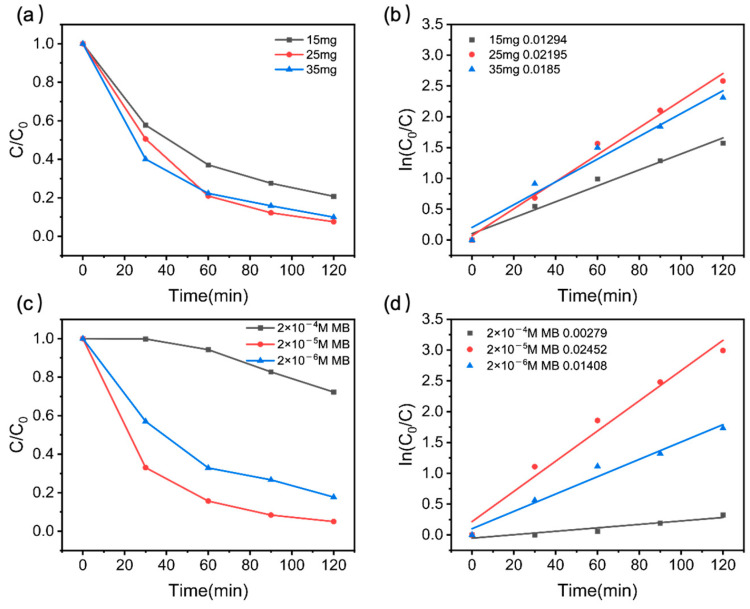
The effect of the amount of BiFeO_3_/Bi_25_FeO_40_ on (**a**) C/C_0_ the concentration changes of MB; (**b**) the reaction rate constant ln(C_0_/C) curves; and the effect of initial concentration of MB on the (**c**) C/C_0_ the concentration changes of MB; (**d**) the reaction rate constant ln(C_0_/C) curves.

The effect of the catalyst amount and the initial concentration of MB on the degradation rate was also examined. Various BiFeO_3_/Bi_25_FeO_40_ catalyst concentrations (15 mg, 25 mg, and 35 mg) alongside initial MB concentrations (2 × 10^−4^, 2 × 10^−5^, and 2 × 10^−6^ M) on photocatalytic performance were tested (see Figure 4). Figure 4a,b illustrate that an increase in catalyst dosage from 15 mg to 25 mg leads to enhanced degradation efficiency as a result of more active sites. However, it was noted that the photocatalytic activity was slightly reduced when the dosage was 30 mg because agglomeration among nanoparticles can easily occur, which reduces the active sites or suppresses the light transmission in the suspension system, leading to a lowered photo-irradiation on the catalyst surface [27,44]. This phenomenon was also informed from Figure 4c,d, which depict the relationship between degradation rate and MB concentration. Notably, as the concentration of MB decreases, the degradation rate typically rises. This occurs because lower MB concentrations provide more active sites on the photocatalyst surface for the degradation reaction, resulting in a higher degradation rate. However, at very low MB concentrations, the degradation efficiency may decline, as a limited number of MB molecules are available for the photocatalytic reaction.

**Figure 5:**
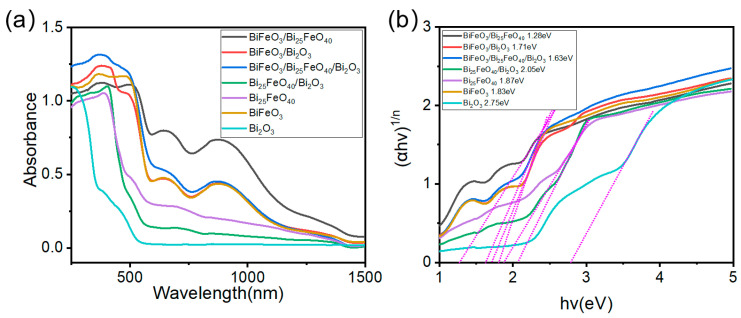
(**a**) UV–vis diffusion reflectance spectra and (**b**) Tauc plots of Bi_2_O_3_, BiFeO_3_, Bi_25_FeO_40_, BiFeO_3_/Bi_25_FeO_40_, BiFeO_3_/Bi_2_O_3_, Bi_25_FeO_40_/Bi_2_O_3_, and BiFeO_3_/Bi_25_FeO_40_/Bi_2_O_3_.

To investigate the mechanisms underlying the enhanced photocatalytic performance of BiFeO_3_-Bi_25_FeO_40_-Bi_2_O_3_ heterojunctions, UV–Vis diffuse reflectance spectroscopy, which is pivotal for evaluating their potential applications in solar energy harvesting, was employed to characterize their optical properties. As depicted in Figure 5a, both BiFeO_3_ and Bi_25_FeO_40_ exhibit significantly stronger visible-light absorption compared to Bi_2_O_3_. BiFeO_3_/Bi_25_FeO_40_ and BiFeO_3_/Bi_25_FeO_40_/Bi_2_O_3_ demonstrate pronounced enhancements in light absorption across the entire visible-light spectrum. In addition, the incorporation of Bi_2_O_3_ into BiFeO_3_ also results in stronger visible-light absorption than pure BiFeO_3_ and Bi_2_O_3_. Differently, the light absorption capability of BiFeO_3_/Bi_25_FeO_40_/Bi_2_O_3_ and Bi_25_FeO_40_/Bi_2_O_3_ is diminished relative to that of Bi_25_FeO_40_ alone but remains superior to that of Bi_2_O_3_. To estimate the band gaps of these materials, we applied Tauc’s equation, which is expressed as (αhυ)^1/n^ = A(hυ-Eg), where α, h, υ, A, and Eg represent the light absorption coefficient, Planck constant, light frequency, constant, and band gap, respectively. The value of n is set to 2, which is appropriate for indirect bandgap semiconductors. The resulting plots, illustrated in Figure 5b, yield band gap values of 2.75 eV for Bi_2_O_3_, 1.83 eV for BiFeO_3_, and 1.87 eV for Bi_25_FeO_40_, aligning well with previously reported experimental and theoretical findings [14,25,31,56,57,58]. The integration of compositions within the BiFeO_3_-Bi_25_FeO_40_-Bi_2_O_3_ system has the potential to induce strains at the interfaces due to variations in their crystal structures. This strain can significantly alter the electronic properties of the materials, affecting the distribution and mobility of charge carriers [59]. Because of this effect, the band gap of the BiFeO_3_/Bi_25_FeO_40_ composite is substantially reduced to 1.28 eV, which enhances its light absorption capability through improved electronic interactions within the heterojunction structure. In the case of the BiFeO_3_/Bi_2_O_3_ composite, the estimated band gap is 1.71 eV, slightly lower than that of BiFeO_3_, thereby facilitating efficient carrier generation. However, the increased band gap of Bi_25_FeO_40_/Bi_2_O_3_ was observed compared to Bi_25_FeO_40_ alone, which can be attributed to crystal lattice mismatch. Additionally, TEM characterizations, as illustrated in Figure 2c, reveal that the incorporation of Bi_2_O_3_ into Bi_25_FeO_40_ generates additional defects, further contributing to the increased band gap.

**Figure 6:**
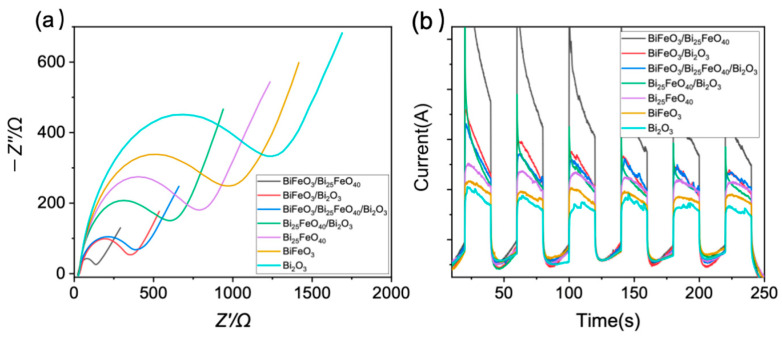
(**a**) EIS measurements and (**b**) photocurrent curves of Bi_2_O_3_, BiFeO_3_, Bi_25_FeO_40_, BiFeO_3_/Bi_25_FeO_40_, BiFeO_3_/Bi_2_O_3_, Bi_25_FeO_40_/Bi_2_O_3_, and BiFeO_3_/Bi_25_FeO_40_/Bi_2_O_3_.

To evaluate the efficiency of the photocatalytic process, we measured the charge transfer resistance of the BiFeO_3_-Bi_25_FeO_40_-Bi_2_O_3_ system (see Figure 6a). It is noted that pure Bi_2_O_3_ demonstrates the highest charge transfer resistance among all the materials tested, and pure phases of Bi_25_FeO_40_ and BiFeO_3_ present higher charge transfer resistance. In contrast, the results of the charge transfer resistance measurements indicate that the BiFeO_3_/Bi_25_FeO_40_ composite exhibits the lowest charge transfer resistance. This suggests that the BiFeO_3_/Bi_25_FeO_40_ heterojunction facilitates the most efficient charge separation and transport kinetics, which can potentially enhance the photocatalytic activity compared to the individual phases of BiFeO_3_ and Bi_25_FeO_40_. The BiFeO_3_/Bi_2_O_3_ composite also shows a relatively low charge transfer resistance, indicating improved charge transfer characteristics compared to the pure BiFeO_3_ and Bi_2_O_3_ phases. In addition, the combinations of BiFeO_3_/Bi_25_FeO_40_/Bi_2_O_3_ and Bi_25_FeO_40_/Bi_2_O_3_ exhibit intermediate levels of charge transfer resistance, suggesting that the addition of Bi_2_O_3_ to the BiFeO_3_/Bi_25_FeO_40_ or Bi_25_FeO_40_ may inhibit the charge transfer kinetics by introducing more complex charge transport channels. This trend in charge transfer resistance is attributed to the varying crystal structures and compositions of the materials, with the BiFeO_3_/Bi_25_FeO_40_ composite likely having the most favorable interface and charge transfer pathways.

These observed trends were further validated through the characterization of transient photocurrent responses, which offer insights into the charge separation efficiency of various BiFeO_3_-Bi_25_FeO_40_-Bi_2_O_3_ heterojunctions. The order of photocurrent response, illustrated in Figure 6b, is as follows: BiFeO_3_/Bi_25_FeO_40_ > BiFeO_3_/Bi_2_O_3_ > BiFeO_3_/Bi_25_FeO_40_/Bi_2_O_3_ > Bi_25_FeO_40_/Bi_2_O_3_ > Bi_25_FeO_40_ > BiFeO_3_ > Bi_2_O_3_. Again, the individual materials (BiFeO_3_, Bi_25_FeO_40_, and Bi_2_O_3_) exhibit lower photocurrent responses compared to these heterojunctions, indicating less efficient charge separation and transport. For the heterojunctions, the BiFeO_3_/Bi_25_FeO_40_ heterojunction demonstrates the highest charge separation efficiency, likely due to the effective transfer of photogenerated charge carriers across the interface. The BiFeO_3_/Bi_2_O_3_ heterojunction also exhibits a relatively high photocurrent response, indicative of good charge separation characteristics. This suggests the combination of BiFeO_3_ and Bi_2_O_3_ likely generates an appropriate band structure that enhances charge carrier separation and transfer. Conversely, the ternary heterojunction BiFeO_3_/Bi_25_FeO_40_/Bi_2_O_3_ shows an intermediate photocurrent response. This behavior may stem from the complex interplay of charge transfer pathways and the presence of multiple interfaces, which can introduce additional scattering or recombination pathways. Additionally, the Bi_25_FeO_40_/Bi_2_O_3_ heterojunction displays a relatively low photocurrent response, potentially due to limited charge transfer dynamics.

**Figure 7:**
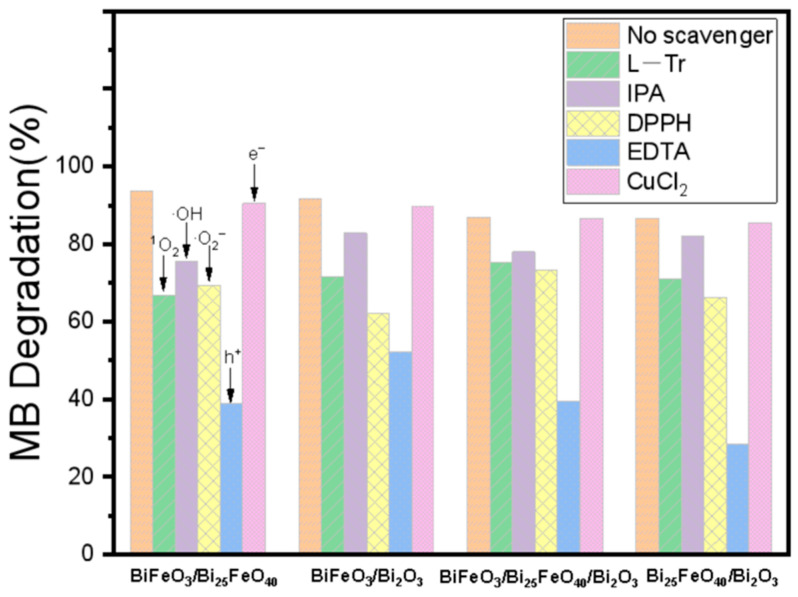
Radical trapping experiments of BiFeO_3_/Bi_25_FeO_40_, BiFeO_3_/Bi_2_O_3_, BiFeO_3_/Bi_25_FeO_40_/Bi_2_O_3,_ and Bi_25_FeO_40_/Bi_2_O_3_. The height of the bars represents the degradation percentage of MB individually.

Through a radical capture experiment, an in-depth study was conducted on the active species involved in the photocatalytic degradation of MB. Ethylene diamine tetraacetic acid (EDTA), CuCl_2_, 2,2-Diphenyl-1-picrylhydrazyl) (DPPH), L-tryptophan (L-Tr), and isopropanol (IPA) are typical scavengers for holes (h^+^), electrons (e^−^), superoxide radical anions (O_2_^−^), singlet oxygen (^1^O_2_), and hydroxyl radicals (·OH), respectively. As shown in Figure 7 and Figure S2, the addition of these scavengers significantly affected the photocatalytic efficiency, except for CuCl_2_. The inclusion of DPPH, L-Tr, and IPA resulted in a certain degree of inhibition of MB degradation. The degradation rates for BiFeO_3_/Bi_25_FeO_40_ were reduced to 42–52% compared to the original rates without a scavenger. Similarly, the degradation rates of BiFeO_3_/Bi_2_O_3_ reduced to 39–70%, those of BiFeO_3_/Bi_25_FeO_40_/Bi_2_O_3_ reduced to 65–76%, and those of Bi_25_FeO_40_/Bi_2_O_3_ reduced to 53–63%, indicating that O_2_^−^, ^1^O_2_, and ·OH are the active species in the photocatalytic degradation of MB. It is noteworthy that the addition of IPA did not result in any significant change in the degradation rate of Bi_25_FeO_40_/Bi_2_O_3_, suggesting that ·OH does not participate in the photocatalytic degradation process of Bi_25_FeO_40_/Bi_2_O_3_. This may be related to the band position, which is discussed in a later section. Following the formation of the heterojunction, the relatively significant difference in the valence band positions between Bi_25_FeO_40_ and Bi_2_O_3_ facilitates the migration of holes to the higher valence band of Bi_2_O_3_ under the influence of the built-in electric field, which weakens the oxidative capability of the heterojunction and hinders the formation of ·OH. This is also corroborated by the relatively low photocatalytic efficiency of the Bi_25_FeO_40_/Bi_2_O_3_ heterojunction and Bi_2_O_3_. More importantly, the addition of EDTA reduced the degradation rates for BiFeO_3_/Bi_25_FeO_40_, BiFeO_3_/Bi_2_O_3_, BiFeO_3_/Bi_25_FeO_40_/Bi_2_O_3_, and Bi_25_FeO_40_/Bi_2_O_3_ to 18%, 29%, 24%, and 17% of their original values, respectively, indicating that holes are the main active species for these four catalysts, participating directly or indirectly in the degradation reaction of MB. Therefore, the photocatalytic degradation of MB over these materials can be described by the following equations:

BiFeO_3_-Bi_25_FeO_40_-Bi_2_O_3_ heterojunction + hv → e^−^ + h^+^ (1)

e^−^ + O_2_ → O_2_^−^ + *OH = ^1^O_2_ + OH^−^ (2)

h^+^ + OH^−^ → ·OH (3)

h^+^(mainly)/O_2_^−^/^1^O_2_/OH + MB → H_2_O + CO_2._ (4)

We experimentally determined the positions of the conduction band and valence band of these materials through UPS measurement (see Figure S3), which allowed us to accurately understand the reaction mechanisms. The valence band position (E_VB_) and conduction band position (E_CB_) relative to the vacuum energy level can be calculated using Equations (5)–(7):

ϕ_s_ = hυ − E_SC_ (5)

E_vb_ = −ϕ_s_ − E_fermi_ (6)

**Figure 8:**
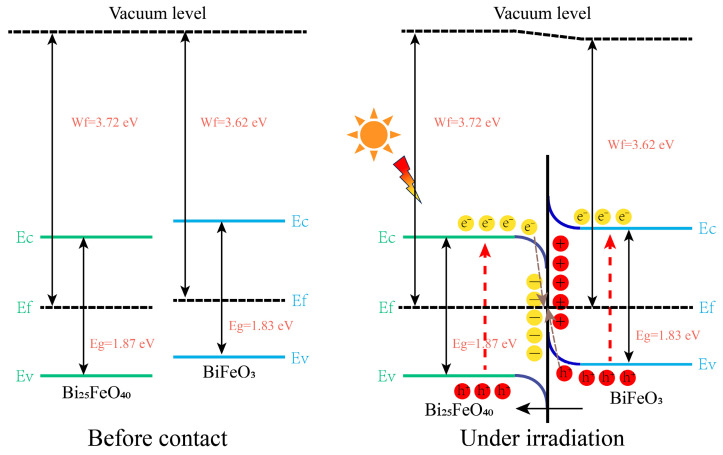
Proposed mechanism for photodegradation of MB by BiFeO_3_/Bi_25_FeO_40_ under solar light.

E_cb_ = E_vb_ + Eg (7)

where ϕ_s_ is the work function of the semiconductor, hυ is related to the photon energy (21.2 eV), E_SC_ is the secondary electron cutoff edge, and E_fermi_ is the Fermi edge. Based on the experimental results and characterization analysis, the reaction mechanisms in the photocatalytic degradation of MB can be understood by examining the band alignment and charge carrier dynamics of the different BiFeO_3_/Bi_25_FeO_40_/Bi_2_O_3_ heterojunction systems. As shown in Figure 8, the energy bands of BiFeO_3_/Bi_25_FeO_40_ show a staggered alignment, where the conduction band (CB) and valence band (VB) energy levels of BiFeO_3_ are higher than those of Bi_25_FeO_40_. Furthermore, because the Fermi level of BiFeO_3_ is higher than that of Bi_25_FeO_40_, electrons will spontaneously transfer from the BiFeO_3_ to the Bi_25_FeO_40_ interface after the heterojunction is formed, generating a built-in electric field that points from BiFeO_3_ to Bi_25_FeO_40_. This leads to the formation of electron depletion and accumulation layers at the interfaces, causing band bending, which conforms to the formation conditions of S-scheme heterojunction. The barriers formed by the band bending and Coulomb repulsion inhibit the transfer of electrons from the CB of BiFeO_3_ to the CB of Bi_25_FeO_40_, while promoting the recombination of electrons in the CB of Bi_25_FeO_40_ with holes in the VB of BiFeO_3_. Under light irradiation, photogenerated electrons are driven from the CB of BiFeO_3_ to Bi_25_FeO_40_ by the electric field and recombine with holes in the VB of Bi_25_FeO_40_ at the heterojunction interface. Meanwhile, electrons in the CB of BiFeO_3_ have stronger reductive capabilities, and holes in the VB of Bi_25_FeO_40_ have higher oxidative capabilities, which are crucial for the oxidation of MB with holes as the main active species and for the involvement in the generation of other radicals. Therefore, the BiFeO_3_/Bi_25_FeO_40_ S-scheme heterojunction not only improves the mobility of photogenerated charge carriers and effectively suppresses electron–hole recombination, but also enhances the involvement of stronger oxidative/reductive holes and electrons in the degradation process, significantly boosting the degradation performance of the BiFeO_3_/Bi_25_FeO_40_ photocatalyst. Moreover, the enhanced charge separation in the BiFeO_3_/Bi_25_FeO_40_ heterojunction is further supported by its significantly higher photocurrent response and smaller charge transfer resistance compared to the other samples. Additionally, the BiFeO_3_/Bi_25_FeO_40_ system demonstrates the strongest light absorption in the visible and near-infrared regions, which contributes to its superior photocatalytic performance.

**Figure 9:**
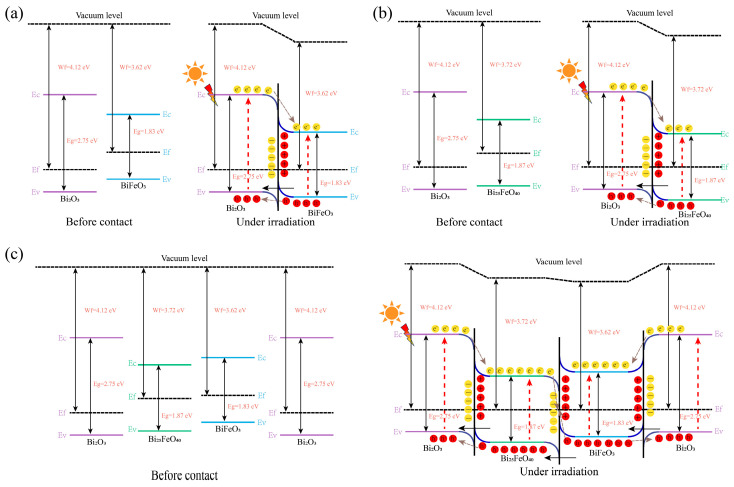
Proposed mechanism for photodegradation of MB by (**a**) BiFeO_3_/Bi_2_O_3_, (**b**) Bi_25_FeO_40_/Bi_2_O_3_, and (**c**) BiFeO_3_/Bi_25_FeO_40_/Bi_2_O_3_ under solar light irradiation.

In contrast, the BiFeO_3_/Bi_2_O_3_ and Bi_25_FeO_40_/Bi_2_O_3_ heterojunctions are classified as Type-II systems (see Figure 9). Since the Fermi levels of BiFeO_3_ and Bi_25_FeO_40_ are both higher than that of Bi_2_O_3_, electrons will spontaneously migrate from BiFeO_3_ or Bi_25_FeO_40_ to Bi_2_O_3_ when forming a heterojunction. At the heterojunction interface, this also results in band bending and the formation of a built-in electric field. Unlike BiFeO_3_/Bi_25_FeO_40_, the band bending in the BiFeO_3_/Bi_2_O_3_ and Bi_25_FeO_40_/Bi_2_O_3_ heterojunctions causes electrons to migrate from the conduction band of Bi_2_O_3_ to the conduction bands of BiFeO_3_ or Bi_25_FeO_40_, while holes migrate from the valence bands of BiFeO_3_ or Bi_25_FeO_40_ to the valence band of Bi_2_O_3_, which aligns with the characteristics of Type-II heterojunctions. However, this separation direction of electrons and holes comes at the cost of reducing the redox ability of the heterojunction. Additionally, due to the presence of electrostatic interactions, the existing photogenerated electron–hole pairs in the original photocatalyst can inhibit the interfacial transfer of electron–hole pairs in other catalysts. Therefore, the electron–hole separation efficiency and redox capabilities of the BiFeO_3_/Bi_2_O_3_ and Bi_25_FeO_40_/Bi_2_O_3_ systems are limited, as evidenced by the reduced photocatalytic degradation rates of MB compared to BiFeO_3_/Bi_25_FeO_40_ (0.02251 min^−1^), along with the inferior photocurrent response and higher charge transfer resistance. Furthermore, Bi_25_FeO_40_/Bi_2_O_3_ has a higher bandgap than BiFeO_3_/Bi_2_O_3_, leading to a poorer visible light response and weaker photocatalytic performance. As for BiFeO_3_/Bi_25_FeO_40_/Bi_2_O_3_, when Bi_2_O_3_ is loaded onto BiFeO_3_/Bi_25_FeO_40_ nanosheets, its bandgap is significantly higher than that of the other two materials, resulting in the formation of a Type-II heterojunction on both sides of the junction (see Figure 9c). Under illumination, the electrons and holes on Bi_2_O_3_ do not effectively separate. Additionally, the relative content of BiFeO_3_/Bi_25_FeO_40_ is reduced, resulting in a moderate photocatalytic degradation rate. Furthermore, the introduction of Bi_2_O_3_ may complicate charge dynamics and induce lattice mismatches or local defects (recombination centers), all of which decrease the charge transfer efficiency among the three materials, while rapid recombination processes further weaken the overall photocatalytic activity.

## 4. Materials and Methods

### 4.1. Synthesis

The bulk BiFeO_3_ and Bi_25_FeO_40_ powders were prepared via a precipitation method in-house, and Bi_2_O_3_ was purchased from Aladdin Chemical Reagent Co., Ltd (Shanghai, China). Bismuth nitrate (Bi(NO_3_)_3_·5H_2_O) and iron nitrate (Fe(NO_3_)_3_·9H_2_O) were used as the precursor materials. For the BiFeO_3_ synthesis, 1.746 g Bi(NO_3_)_3_·5H_2_O and 1.454 g Fe(NO_3_)_3_·9H_2_O were dissolved in 50 mL aqueous solution with a 1:1 molar ratio. Then 2 M sodium hydroxide (NaOH) solution was slowly added to the precursor solution to adjust the pH to 10, facilitating ferritization. The resulting precipitate was centrifuged and washed with deionized water four times to remove residual ions. The washed precipitate was then dried in an oven at 80 °C overnight. Finally, the dried powders were placed in a tube furnace and heated at 700 °C for 1 h under a 10% O_2_ in Ar atmosphere to obtain BiFeO_3_. To synthesize Bi_25_FeO_40_, the nominal amounts used were 2.42535 g of Bi(NO_3_)_3_·5H_2_O and 0.0808 g of Fe(NO_3_)_3_·9H_2_O, ensuring a molar ratio of Bi to Fe of 25:1. The subsequent steps followed the same procedure as those used for synthesizing BiFeO_3_.

For the synthesis of heterojunctions, all BiFeO_3_-Bi_25_FeO_40_-Bi_2_O_3_ heterojunctions were prepared using a wetness impregnation method. In the case of BiFeO_3_/Bi_2_O_3_, a homogeneous solution was prepared by dissolving 1.551 g of Bi(NO_3_)_3_·5H_2_O and 1.291 g of Fe(NO_3_)_3_·9H_2_O, maintaining a 1:1 molar ratio of Bi to Fe, in 5 mL of deionized water. In this typical synthesis, Bi_2_O_3_ comprises 50 wt% of the BiFeO_3_/Bi_2_O_3_ mixture. Thus, 1 g of Bi_2_O_3_ powder was added to the precursor solution and mixed homogeneously. The resulting paste was dried in an oven at 80 °C overnight and subsequently placed in a tube furnace, where it was heated to 600 °C in a 10% O_2_ in Ar atmosphere for one hour to form the BiFeO_3_/Bi_2_O_3_ heterojunction materials. When the pretreatment temperature was increased to 700 °C and 750 °C, BiFeO_3_/Bi_25_FeO_40_/Bi_2_O_3_ and BiFeO_3_/Bi_25_FeO_40_ were produced, respectively. Additionally, Bi_25_FeO_40_/Bi_2_O_3_ was synthesized using a similar method by combining 2.021 g of Bi(NO_3_)_3_·5H_2_O, 0.067 g of Fe(NO_3_)_3_·9H_2_O, and 1 g of Bi_2_O_3_, with the same pretreatment condition of 600 °C in a 10% O_2_ in Ar atmosphere for one hour.

It is noted that all samples have good reproducibility in their synthesis, and each sample can be synthesized in amounts of approximately 20 g. After thermal treatment, all samples were allowed to cool naturally to room temperature and were then collected for further characterization and photocatalytic evaluation.

### 4.2. Characterization

All prepared heterojunction samples, as well as single Bi_2_O_3_, BiFeO_3_, and Bi_25_FeO_40_ samples, were characterized. The crystal structures of the synthesized materials were analyzed by X-ray diffraction (XRD) using a Ultima IV diffractometer (Rigaku Corporation, Tokyo, Japan). The XRD patterns were recorded in the 2θ range of 10–90° with a step size of 0.02° and a scan rate of 2°/min using Cu Kα radiation (λ = 1.5406 Å). The morphologies of the heterojunctions were examined using transmission electron microscopy (TEM, JEM-2100F, JEOL Ltd, Tokyo, Japan). The samples were dispersed in ethanol and drop-cast onto copper grids coated with a thin carbon film. High-resolution TEM (HRTEM) images were obtained to analyze the lattice fringes and interplanar spacing. The chemical composition and oxidation states of the elements in the heterojunction were investigated by X-ray photoelectron spectroscopy (XPS, Shimadzu/Krayos AXIS Ultra DLD, Kyoto, Japan). The XPS spectra were recorded using a monochromatic Al Kα X-ray source (hν = 1486.6 eV) with a pass energy of 50 eV and a step size of 0.1 eV.

The optical properties of materials were investigated using ultraviolet–visible diffuse reflectance spectroscopy (UV-vis DRS) on a Shimadzu UV-3600IPLUS spectrophotometer from Japan. The diffuse reflectance spectra were recorded in the wavelength range of 200 to 800 nm, and the Kubelka–Munk function was applied to convert the reflectance data into absorbance data. Ultraviolet photoelectron spectroscopy (UPS) was conducted using a Thermo Fisher Scientific ESCALAB XI+ to determine the features of the electronic structure of materials with a bias potential of −5 eV. In addition, electrochemical impedance spectroscopy (EIS) and transient photocurrent measurements were conducted using a designated electrochemical workstation. The EIS measurements were performed in a three-electrode configuration, with the heterojunction serving as the working electrode, a platinum foil as the counter electrode, and a saturated calomel electrode (SCE) as the reference electrode. The EIS spectra were recorded over a frequency range of 0.01 Hz to 100 kHz, with an alternating current amplitude of 10 mV. The transient photocurrent response of the heterojunction was measured under a 300 W PLS-SXE300BF xenon lamp with a bias potential of 0.2 V.

### 4.3. Photocatalytic Performance Test

The photocatalytic properties of BiFeO_3_-Bi_25_FeO_40_-Bi_2_O_3_ heterojunctions were investigated in the degradation of MB and phenol with comparisons to as-prepared single materials (BiFeO_3_, Bi_25_FeO_40_, and Bi_2_O_3_). Typically, for the visible-light photocatalysis, a 300 W Xe lamp with a wavelength above 420 nm was utilized. Before running the photocatalytic degradation, a 25 mg photocatalyst was incubated in a cuvette with 50 mL MB solution (2 × 10^−5^ M) or phenol solution (40 mg/L) for one hour in the dark to reach an adsorption–desorption equilibrium. The UV-vis absorption spectra of MB and phenol during the adsorption–desorption phase were taken at 0, 30, and 60 min for reference (see Figure S1). During the photocatalytic degradation process, the efficiency was monitored every 30 min through UV–visible absorption spectroscopy, and each test was repeated at least three times. Furthermore, recycling experiments were performed following the same protocol. After each experimental run, the utilized photocatalyst was centrifugated, followed by washing with deionized water and ethanol, and was subsequently dried for two hours in preparation for subsequent trials. The overall efficiency percentage was determined following Formula (8).

(C_0_ − C)/C_0_ × 100% = (A_0_ − A)/A_0_ × 100% (8)

where C_0_ refers to the initial concentration of the probe molecules, while A_0_ denotes their initial absorbance. Conversely, C represents the remaining concentration, and A indicates their absorbance at that point [60].

Radical trapping experiments were conducted to elucidate the mechanisms underlying photocatalytic degradation processes. Various reagents were employed to scavenge specific reactive species: EDTA was utilized to capture holes (h^+^), CuCl_2_ was used for electrons (e^−^), isopropyl alcohol (IPA) was used to target hydroxyl radicals (⋅OH), L-Tryptophan (L-Tr) was used for singlet oxygen (^1^O_2_), and 2,2-Diphenyl-1-picrylhydrazyl (DPPH) was used for superoxide radicals (⋅O_2_^−^) [61,62,63].

## 5. Conclusions

Developing efficient and cost-effective photocatalysts remains a critical challenge for environmental remediation and solar energy conversion. In this work, we successfully prepared three binary heterojunctions (BiFeO_3_/Bi_25_FeO_40_, BiFeO_3_/Bi_2_O_3_, and Bi_25_FeO_40_/Bi_2_O_3_) as well as a ternary BiFeO_3_/Bi_25_FeO_40_/Bi_2_O_3_ heterojunction using a simple one-pot method. Among these materials, the BiFeO_3_/Bi_25_FeO_40_ heterojunction demonstrated an impressive photocatalytic efficiency of 93.68% and 83.6% for the degradation of MB and phenol under visible light, surpassing both BiFeO_3_ and Bi_25_FeO_40_, as well as most performances reported by others. The other heterojunctions also showed enhanced catalytic performance compared to their counterparts. These trends align with the insights derived from characterizations of light absorption ability, charge separation, and photocurrent response relative to the composition of the heterojunctions. Further investigation of the reaction mechanism suggests enhanced performance of the BiFeO_3_/Bi_25_FeO_40_ heterojunction is attributed to its S-scheme heterojunction, which promotes efficient charge separation and redox capabilities. In contrast, the Type-II systems of BiFeO_3_/Bi_2_O_3_ and Bi_25_FeO_40_/Bi_2_O_3_ heterojunctions resulted in reduced photocatalytic activity compared to BiFeO_3_/Bi_25_FeO_40_. In addition, when Bi_2_O_3_ is integrated with BiFeO_3_/Bi_25_FeO_40_ nanosheets, the electrons and holes are not separated effectively, leading to diminished photocatalytic activity. In future research, further control of the morphology and size, as well as modifications of heterojunction composition, will be conducted to yield even greater performance improvements and provide a deeper understanding of the underlying reaction mechanism.

## Figures and Tables

**Table 1:** Comparison, with reported literature, of photocatalyst activity of prepared catalysts in the degradation of MB and phenol in this work.

Catalyst	Organic Molecule	K (min−1)	K/mcatalyst (min^−1^·g^−1^)	Synthesis Methods	Degradation Efficiency	Light Source	Ref.
BiFeO_3_/ Bi_25_FeO_40_	MB phenol	0.0225 0.0159	0.900 0.636	wetness impregnation	93.68% @120 min	300 W Xe lamp (λ ≥ 420 nm)	This work
BiFeO_3_/ Bi_2_O_3_	MB phenol	0.0208 0.0051	0.832 0.204	wetness impregnation	91.86% @120 min	300 W Xe lamp (λ ≥ 420 nm)	This work
BiFeO_3_/ Bi_25_FeO_40_/Bi_2_O_3_	MB phenol	0.0170 0.0047	0.678 0.188	wetness impregnation	87.03% @120 min	300 W Xe lamp (λ ≥ 420 nm)	This work
Bi_25_FeO_40_/ Bi_2_O_3_	MB phenol	0.0168 0.0040	0.673 0.16	wetness impregnation	86.63% @120 min	300 W Xe lamp (λ ≥ 420 nm)	This work
BiFeO_3_	MB phenol	0.0157 0.0038	0.633 0.152	precipitation	83.85% @120 min	300 W Xe lamp (λ ≥ 420 nm)	This work
Bi_25_FeO_40_	MB phenol	0.0158 0.0038	0.607 0.152	precipitation	84.16% @120 min	300 W Xe lamp (λ ≥ 420 nm)	This work
Bi_2_O_3_	MB phenol	0.0137 0.0016	0.548 0.064	precipitation	80.05% @120 min	300 W Xe lamp (λ ≥ 420 nm)	This work
Cr doped CeO_2_	MB	0.0027	0.137	deposition precipitation	59% @100 min	UV irradiation	[41]
MoO_3_/BiVO_4_	MB	0.0036	0.18	co-precipitation	76% @120 min	visible-light (λ ≥ 420 nm)	[42]
(Sr_0.6_Bi_0.305_)_2_Bi_2_O_7_/TiO_2_	MB	0.0342	0.171	hydrothermal method	95.5% @120 min	300 W Xe lamp (λ > 400 nm)	[43]
Fe_3_O_4_/SiO_2_/MnO_2_/BiOBr	MB	0.013	0.867	solvothermal method	95.23% @150 min	400 W metal halide lamp	[44]
AgBr/Bi_2_WO_6_	MB	0.0785	0.393	precipitation-deposition	100% @120 min	500 W Xe-arc lamp (λ ≥ 420 nm)	[45]
ZIF-8	MB	0.017	0.68	slow evaporation	82.3% @120 min	500 W Hg lamp	[46]
(Yb, N)-TiO_2_	MB	0.0091	0.061	sol–gel method	93.55% @5 h	30W fluorescent lamp (400–750 nm)	[47]
g-C_3_N_4_-RGO-TiO_2_	MB	0.0137	0.274	Liquid precipitation	92% @3 h	300 W Xe lamp (λ ≥ 420 nm)	[48]
TiO_2_	MB	0.0142	0.356	hydrothermal method	93.92% @60 min	250 W mercury lamp	[49]
Bi/Bi_4_Ti_3_O_12_	MB	0.0077	0.773	in-situ reduction	87.2% @120 min	Xe lamp	[50]
Bi_2_O_3_/(BiO)_2_CO_3_/Bi_2_MoO6	phenol	0.0162	0.081	solvothermal	98.8% @180 min	400 W halogen lamp (λ > 420 nm)	[51]
Fe_3_O_4_-ZnO	phenol	0.0039	0.06	precipitation	82.3% @150 min	MSR 575/2 metal halide lamp	[52]
ZnO-Graphene /TiO_2_-Graphene	phenol	0.0378	-	deposition	100% @60 min	1000 W Xe arc lamp	[53]
Bi_2_O_3_-Bi_2_O_2.33_	phenol	-	-	co-precipitation	95% @60 min	500 W Xe lamp (λ ≥ 400 nm)	[54]
TiO_2_-AC	phenol	0.0139	0.278	-	100% @180 min	a high-pressure mercury lamp	[55]

## Data Availability

Data will be made available on request.

## Supplementary Materials

The following supporting information can be downloaded at: https://www.mdpi.com/article/10.3390/ijms26010196/s1.

## References

[1] Rueda-Marquez J.J., Levchuk I., Fernández Ibañez P., Sillanpää M. (2020). A Critical Review on Application of Photocatalysis for Toxicity Reduction of Real Wastewaters. J. Clean. Prod..

[2] Liu S.-Q. (2012). Magnetic Semiconductor Nano-Photocatalysts for the Degradation of Organic Pollutants. Environ. Chem. Lett..

[3] Anjaneyulu B., Chauhan V., Vashisth C., Yogita, Chinmay, Raghav N. (2024). Revolutionizing Industrial Wastewater Treatment: MXenes Conquer Organic Pollutants in a Paradigm Shifting Breakthrough towards Sustainability. Chem. Eng. J..

[4] Wang C.-Y., Zhang X., Yu H.-Q. (2023). Bismuth Oxyhalide Photocatalysts for Water Purification: Progress and Challenges. Coord. Chem. Rev..

[5] Chen D., Cheng Y., Zhou N., Chen P., Wang Y., Li K., Huo S., Cheng P., Peng P., Zhang R. (2020). Photocatalytic Degradation of Organic Pollutants Using TiO_2_-Based Photocatalysts: A Review. J. Clean. Prod..

[6] Kantawong T., Lee V.S., Nimmanpipug P. (2016). Anatase TiO_2_ (101) and Wurtzite ZnO (001) Modified Polymer for Visible Light-Photocatalytic Efficiency Enhancement. Integr. Ferroelectr..

[7] Xiao H., Pei H., Liu J., Cui J., Jiang B., Hou Q., Hu W. (2012). Fabrication, Characterization, and Photocatalysis of GaN–Ga_2_O_3_ Core-Shell Nanoparticles. Mater. Lett..

[8] Shi X., Li J., Zhu J., Yang Y., Wang X., Zhu B., Xu L. (2021). Study of Composite TiN/Ag/Cu_2_O/TiO_2_ with Improved Photocatalytic Performance. PAC.

[9] Wang Q. (2020). Morphology Regulated Bi_2_WO_6_ Nanoparticles on TiO_2_ Nanotubes by Solvothermal Sb^3+^ Doping as Effective Photocatalysts for Wastewater Treatment. Electrochim. Acta.

[10] Majumder S., Quang N.D., Thi Hien T., Chinh N.D., Yang H., Kim C., Kim D. (2021). Nanostructured β-Bi_2_O_3_/PbS Heterojunction as Np-Junction Photoanode for Enhanced Photoelectrochemical Performance. J. Alloys Compd..

[11] Nasr M., Eid C., Habchi R., Miele P., Bechelany M. (2018). Recent Progress on Titanium Dioxide Nanomaterials for Photocatalytic Applications. ChemSusChem.

[12] Yan Y., Zhou Z., Cheng Y., Qiu L., Gao C., Zhou J. (2014). Template-Free Fabrication of α- and β-Bi_2_O_3_ Hollow Spheres and Their Visible Light Photocatalytic Activity for Water Purification. J. Alloys Compd..

[13] Xu J., Wang W., Sun S., Wang L. (2012). Enhancing Visible-Light-Induced Photocatalytic Activity by Coupling with Wide-Band-Gap Semiconductor: A Case Study on Bi_2_WO_6_/TiO_2_. Appl. Catal. B Environ..

[14] Xia P., Song Y.-J., Liu Y.-Z., Long M.-X., Yang C., Zhang X.-Y., Zhang T. (2024). Advances in the Optical and Electronic Properties and Applications of Bismuth-Based Semiconductor Materials. J. Mater. Chem. C.

[15] Li Q.-Y., Zhao Z.-Y. (2015). Interfacial Properties of α/β-Bi_2_O_3_ Homo-Junction from First-Principles Calculations. Phys. Lett. A.

[16] Li Z., Shen Y., Guan Y., Hu Y., Lin Y., Nan C.-W. (2014). Bandgap Engineering and Enhanced Interface Coupling of Graphene–BiFeO_3_ Nanocomposites as Efficient Photocatalysts under Visible Light. J. Mater. Chem. A.

[17] Wang N., Luo X., Han L., Zhang Z., Zhang R., Olin H., Yang Y. (2020). Structure, Performance, and Application of BiFeO_3_ Nanomaterials. Nano Micro Lett..

[18] Venugopal V., Balaji D., Preeyanghaa M., Moon C.J., Neppolian B., Muthusamy G., Theerthagiri J., Madhavan J., Choi M.Y. (2023). Synergistic Combination of BiFeO_3_ Nanorods and CeVO_4_ Nanoparticles for Enhanced Visible Light Driven Photocatalytic Activity. Alex. Eng. J..

[19] Benyoussef M., Saitzek S., Rajput N.S., Courty M., El Marssi M., Jouiad M. (2022). Experimental and Theoretical Investigations of Low-Dimensional BiFeO_3_ System for Photocatalytic Applications. Catalysts.

[20] Orudzhev F.F., Alikhanov N.M.-R., Rabadanov M.K., Ramazanov S.M., Isaev A.B., Gadzhimagomedov S.K., Aliyev A.S., Abdullaev V.R. (2018). Synthesis and Study of The Properties of Magnetically Separable Nanophotocatalyst BiFeO_3_. Chem. Probl..

[21] Senthilkumar N., Selvaraj Y., Eswaramoorthy N., Pandiaraj S., Alibrahim K.A., Alodhayb A.N. (2024). Optimizing Photocatalytic and Supercapacitive Performance by β-Bi_2_WO_6_@BiFeO_3_ Modification with PVDF Polymer Based Nanocomposites. Colloids Surf. A Physicochem. Eng. Asp..

[22] De Góis M.M., De Paiva Araújo W., Da Silva R.B., Da Luz G.E., Soares J.M. (2019). Bi_25_FeO_40_ − Fe_3_O_4_ − Fe_2_O_3_ Composites: Synthesis, Structural Characterization, Magnetic and UV–Visible Photocatalytic Properties. J. Alloys Compd..

[23] Zhang L., Zou Y., Song J., Pan C.-L., Sheng S.-D., Hou C.-M. (2016). Enhanced Photocatalytic Activity of Bi_25_FeO_40_–Bi_2_WO_6_ Heterostructures Based on the Rational Design of the Heterojunction Interface. RSC Adv..

[24] Wang G., Cheng D., He T., Hu Y., Deng Q., Mao Y., Wang S. (2019). Enhanced Visible-Light Responsive Photocatalytic Activity of Bi_25_FeO_40_/Bi_2_Fe_4_O_9_ Composites and Mechanism Investigation. J. Mater. Sci. Mater. Electron..

[25] Wang Y., Xu C., Yan L., Li J. (2023). Synthesis of BiFeO_3_/Bi_25_FeO_40_ Heterojunction Structure and Precise Adjustment of Forbidden Band Width. Mater. Chem. Phys..

[26] Li X., Qiu Y., Zhu Z., Zhang H., Yin D. (2022). Novel Recyclable Z-Scheme g-C_3_N_4_/Carbon Nanotubes/Bi_25_FeO_40_ Heterostructure with Enhanced Visible-Light Photocatalytic Performance towards Tetracycline Degradation. Chem. Eng. J..

[27] Sun A., Chen H., Song C., Jiang F., Wang X., Fu Y. (2013). Magnetic Bi_25_FeO_40_-Graphene Catalyst and Its High Visible-Light Photocatalytic Performance. RSC Adv..

[28] Huang Y., Nengzi L., Li X., Meng L., Song Q., Cheng X. (2020). Fabrication of Cu_2_O/Bi_25_FeO_40_ Nanocomposite and Its Enhanced Photocatalytic Mechanism and Degradation Pathways of Sulfamethoxazole. Mater. Sci. Semicond. Process..

[29] Wang Y., He Y., Li T., Cai J., Luo M., Zhao L. (2012). Photocatalytic Degradation of Methylene Blue on CaBi_6_O_10_/Bi_2_WO_6_ Composites under Visible Light. Chem. Eng. J..

[30] Kim D., Jung D. (2017). Enhancement of Photocatalytic Activity over Bi_2_O_3_/Black-BiOCl Heterojunction. Chem. Phys. Lett..

[31] Lu H., Hao Q., Chen T., Zhang L., Chen D., Ma C., Yao W., Zhu Y. (2018). A High-Performance Bi_2_O_3_/Bi_2_SiO_5_ p-n Heterojunction Photocatalyst Induced by Phase Transition of Bi_2_O_3_. Appl. Catal. B Environ..

[32] Shi Y., Xiao M., Luo L., Zhang Y., Wang S., Chen Y., Long Y., Li L., Jiang F. (2018). Bi_2_WO_6_/BiFeO_3_ Heterostructure: Preparation, Characterization, and Photocatalytic Activity. Chem. Pap..

[33] Bernardo M.S. (2014). Synthesis, Microstructure and Properties of BiFeO_3_-Based Multiferroic Materials: A Review. Bol. Soc. Esp. Ceram. Vidr..

[34] Jiang L., Yuan X., Zeng G., Liang J., Chen X., Yu H., Wang H., Wu Z., Zhang J., Xiong T. (2018). In-Situ Synthesis of Direct Solid-State Dual Z-Scheme WO_3_/g-C_3_N_4_/Bi_2_WO_6_ Photocatalyst for the Degradation of Refractory Pollutant. Appl. Catal. B Environ..

[35] Liu Y., Guo H., Zhang Y., Tang W., Cheng X., Li W. (2018). Heterogeneous Activation of Peroxymonosulfate by Sillenite Bi_25_FeO_40_: Singlet Oxygen Generation and Degradation for Aquatic Levofloxacin. Chem. Eng. J..

[36] Xu J., Bian Z., Xin X., Chen A., Wang H. (2018). Size Dependence of Nanosheet BiVO_4_ with Oxygen Vacancies and Exposed {0 0 1} Facets on the Photodegradation of Oxytetracycline. Chem. Eng. J..

[37] Lopes O.F., Carvalho K.T.G., Avansi W., Ribeiro C. (2017). Growth of BiVO_4_ Nanoparticles on a Bi_2_WO_6_ Surface: Effect of Heterojunction Formation on Visible Irradiation-Driven Catalytic Performance. J. Phys. Chem. C.

[38] Wu C.-H., Kuo C.-Y., Dong C.-D., Chen C.-W., Lin Y.-L., Chen W.-M. (2019). Synthesis of Bi_2_WO_6_/BiVO_4_ Heterojunction with Enhanced Photocatalytic Activity via Single-Step Hydrothermal Method. Desalination Water Treat..

[39] Wei Y.-L. (2021). Porous and Visible-Light-Driven p–n Heterojunction Constructed by Bi_2_O_3_ Nanosheets and WO_3_ Microspheres with Enhanced Photocatalytic Performance. Sep. Purif. Technol..

[40] Adhikari S.P. (2015). Visible-Light-Driven Bi_2_WO_6_/WO_3_ Composites with Enhanced Photocatalytic Activity. RSC Adv..

[41] Habib I.Y., Burhan J., Jaladi F., Lim C.M., Usman A., Kumara N.T.R.N., Tsang S.C.E., Mahadi A.H. (2021). Effect of Cr Doping in CeO_2_ Nanostructures on Photocatalysis and H_2_O_2_ Assisted Methylene Blue Dye Degradation. Catal. Today.

[42] Liaqat M., Khalid N.R. (2021). Efficient Photocatalysis Performance and Recyclability of MoO_3_/BiVO_4_ Heterostructure under Visible Light. Appl. Nanosci..

[43] Wang X., Hu C., An H., Zhu D., Zhong Y., Wang D., Tang C., Sun L., Zhou H. (2021). Photocatalytic Removal of MB and Hydrogen Evolution in Water by (Sr_0.6_Bi_0.305_)_2_Bi_2_O_7_/TiO_2_ Heterostructures under Visible-Light Irradiation. Appl. Surf. Sci..

[44] Ma M., Yang Y., Chen Y., Ma Y., Lyu P., Cui A., Huang W., Zhang Z., Li Y., Si F. (2021). Photocatalytic Degradation of MB Dye by the Magnetically Separable 3D Flower-like Fe_3_O_4_/SiO_2_/MnO_2_/BiOBr-Bi Photocatalyst. J. Alloys Compd..

[45] Wang D., Guo L., Zhen Y., Yue L., Xue G., Fu F. (2014). AgBr Quantum Dots Decorated Mesoporous Bi_2_WO_6_ Architectures with Enhanced Photocatalytic Activities for Methylene Blue. J. Mater. Chem. A.

[46] Jing H.-P., Wang C.-C., Zhang Y.-W., Wang P., Li R. (2014). Photocatalytic Degradation of Methylene Blue in ZIF-8. RSC Adv..

[47] Zhang J., Xu L.J., Zhu Z.Q., Liu Q.J. (2015). Synthesis and Properties of (Yb, N)-TiO_2_ Photocatalyst for Degradation of Methylene Blue (MB) under Visible Light Irradiation. Mater. Res. Bull..

[48] Wu F., Li X., Liu W., Zhang S. (2017). Highly Enhanced Photocatalytic Degradation of Methylene Blue over the Indirect All-Solid-State Z-Scheme g-C_3_N_4_-RGO-TiO_2_ Nanoheterojunctions. Appl. Surf. Sci..

[49] Gao W., Li Y., Zhao J., Zhang Z., Tang W., Wang J., Wu Z. (2023). Photocatalytic Degradation of Methylene Blue from Aqueous Solutions by rGO/TiO_2_ Nanocomposites. Water Air Soil Pollut..

[50] Xu Q., Zhang Y., Lu M., Gan B., Zhang K., Lu D., Lin S., Liu L., Qin Y. (2024). Excellent Piezo-Photocatalytic Performance of Plasmonic Bi/Bi_4_Ti_3_O_12_ Heterojunction Synthesized by in-Situ Reduction. Surf. Interfaces.

[51] Shen H., Yang C., Xue W., Hao L., Wang D., Fu F., Sun Z. (2023). Construction of Ternary Bismuth-Based Heterojunction by Using (BiO)_2_CO_3_ as Electron Bridge for Highly Efficient Degradation of Phenol. Chem. A Eur. J.

[52] Feng X., Guo H., Patel K., Zhou H., Lou X. (2014). High Performance, Recoverable Fe_3_O_4_ZnO Nanoparticles for Enhanced Photocatalytic Degradation of Phenol. Chem. Eng. J..

[53] Malekshoar G., Pal K., He Q., Yu A., Ray A.K. (2014). Enhanced Solar Photocatalytic Degradation of Phenol with Coupled Graphene-Based Titanium Dioxide and Zinc Oxide. Ind. Eng. Chem. Res..

[54] Peng Y., Wang K.K., Liu T., Xu J., Xu B.G. (2017). Synthesis of One-Dimensional Bi_2_WO_6_-Bi_2_O_2.33_ Heterojunctions with High Interface Quality for Enhanced Visible Light Photocatalysis in Degradation of High-Concentration Phenol and MO Dyes. Appl. Catal. B Environ..

[55] Matos J., Laine J., Herrmann J.-M. (1998). Synergy Effect in the Photocatalytic Degradation of Phenol on a Suspended Mixture of Titania and Activated Carbon. Appl. Catal. B Environ..

[56] Sharma R., Khanuja M., Sharma S.N., Sinha O.P. (2017). Reduced Band Gap & Charge Recombination Rate in Se Doped α-Bi_2_WO_6_ Leads to Enhanced Photoelectrochemical and Photocatalytic Performance: Theoretical & Experimental Insight. Int. J. Hydrogen Energy.

[57] Song Y.-J., Xia P., Zhang X.-Y., Zhang T. (2024). Systematic Investigation on the Rational Design and Optimization of Bi-Based Metal Oxide Semiconductors in Photocatalytic Applications. Nanotechnology.

[58] Satar N.S.A., Aziz A.W., Yaakob M.K., Yahya M.Z.A., Hassan O.H., Kudin T.I.T., Kaus N.H.M. (2016). Experimental and First-Principles Investigations of Lattice Strain Effect on Electronic and Optical Properties of Biotemplated BiFeO_3_ Nanoparticles. J. Phys. Chem. C.

[59] Liu J., Zhang J. (2020). Nanointerface Chemistry: Lattice-Mismatch-Directed Synthesis and Application of Hybrid Nanocrystals. Chem. Rev..

[60] Wang S.-J., Zhang X.-Y., Su D., Yan X., Zhou H.-L., Xue X.-M., Wang Y.-F., Zhang T. (2022). Enhanced Photocatalytic Reactions via Plasmonic Metal-Semiconductor Heterostructures Combing with Solid-Liquid-Gas Interfaces. Appl. Catal. B Environ..

[61] Wang Q. (2024). Construction of Z-Scheme Bi_2_O_3_/CeO_2_ Heterojunction for Enhanced Photocatalytic Capacity of TiO_2_ NTs. Spectrochim. Acta Part A Mol. Biomol. Spectrosc..

[62] Wang Q., Zhao Y., Zhang Z., Liao S., Deng Y., Wang X., Ye Q., Wang K. (2023). Hydrothermal Preparation of Sn_3_O_4_/TiO_2_ Nanotube Arrays as Effective Photocatalysts for Boosting Photocatalytic Dye Degradation and Hydrogen Production. Ceram. Int..

[63] Wang Q., Zhu S., Zhao S., Li C., Wang R., Cao D., Liu G. (2022). Construction of Bi-Assisted Modified CdS/TiO_2_ Nanotube Arrays with Ternary S-Scheme Heterojunction for Photocatalytic Wastewater Treatment and Hydrogen Production. Fuel.

