# Supplementary material for: A Simple One-Pot Method for the Synthesis of BiFeO3/Bi25FeO40 Heterojunction for High-Performance Photocatalytic Degradation Applications
Source: Int J Mol Sci. 2024 Dec 29;26(1):196. doi: 10.3390/ijms26010196 (PMC11719736; doi:10.3390/ijms26010196)
Supplement: Supplementary file 1 [file ijms-26-00196-s001.zip › ijms-3347200-supplementary.pdf]

A Simple One-Pot Method for the Synthesis of $BiFeO_3/Bi_{25}FeO_{40}$ Heterojunction for High-performance Photocatalytic Degradation Applications

Yuan-Jun Song [1,2], Xiao-Ying Bi [1,2], Peng Xia [1,2], Fei Sun [1,2], Ze-Xian Chen [1,2], Xiao-Yang Zhang [1,2,3] and Tong Zhang [1,2,3,*]

[1]Joint International Research Laboratory of Information Display and Visualization, School of Electronic Science and Engineering, Southeast University, Nanjing, Jiangsu 210096, People's Republic of China

[2]Suzhou Key Laboratory of Metal Nano-Optoelectronic Technology, Southeast University Suzhou Campus, Suzhou, Jiangsu 215123, People's Republic of China

[3]Key Laboratory of Micro-Inertial Instrument and Advanced Navigation Technology, Ministry of Education, and School of Instrument Science and Engineering, Southeast University, Nanjing, Jiangsu210096, People's Republic of China

**Correspondence: tzhang@seu.edu.cn;*

Contents

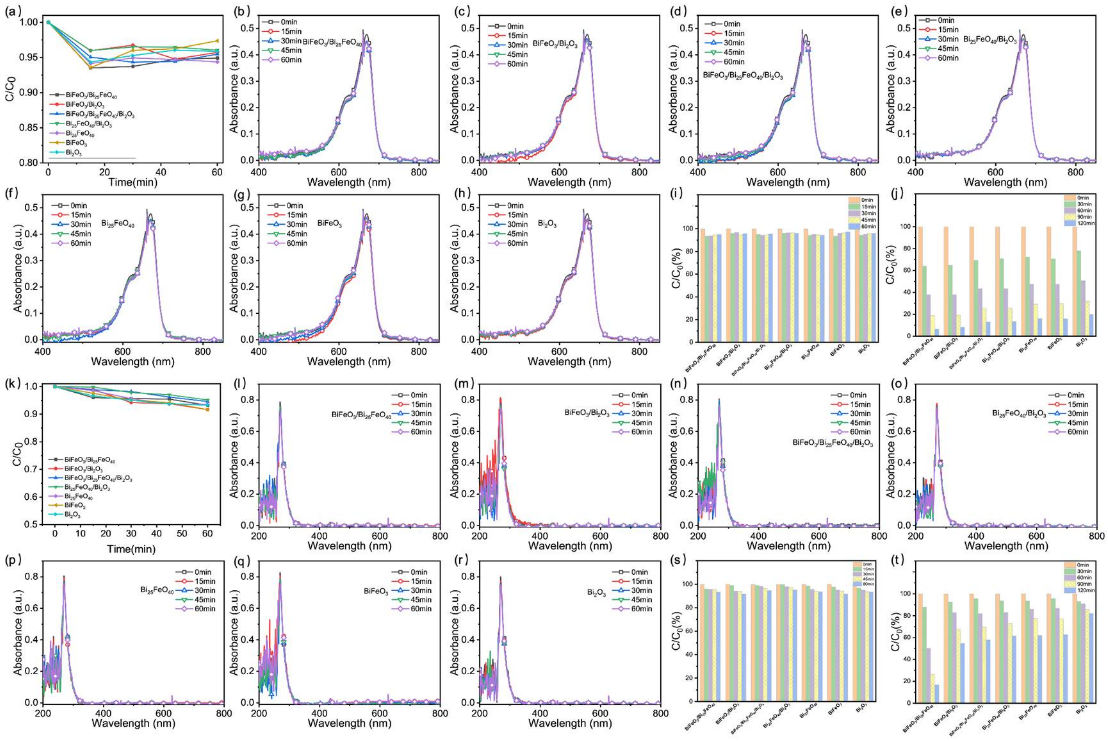

Figure S1. (a) The concentration of MB changes and (b-h) corresponding UV-vis absorption spectra of MB during the adsorption-desorption phase as a function of dark time over $BiFeO_3/Bi_{25}FeO_{40}$, $BiFeO_3/Bi_2O_3$, $Bi_{25}FeO_{40}/Bi_2O_3$, $BiFeO_3/Bi_{25}FeO_{40}/Bi_2O_3$, Bi_2O_3, $BiFeO_3$, and $Bi_{25}FeO_{40}$; (i) the bars for the C/C_0 (%) of MB during the adsorption-desorption phase as a function of time; (j) the bars for the C/C_0 (%) of MB after 60 mins dark as a function of light irradiation time, using the adsorption capacity at 60 mins of dark as the 0 time light irradiation (C/C_0 = 100%). (k)-(t) are the corresponding figures in the same order for phenol degradation tests.

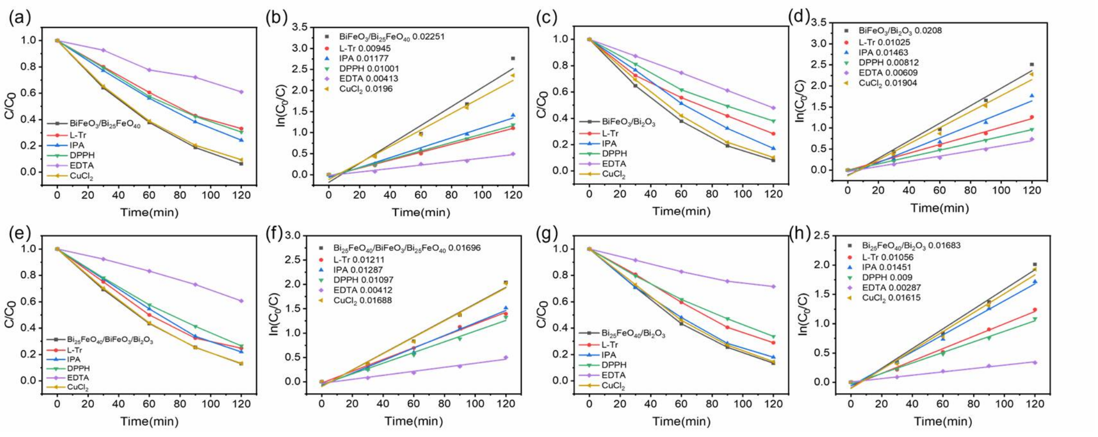

Figure S2. Radicals trapping experiments of the degradation performance as a function time over (a) (b) $BiFeO_3/Bi_{25}FeO_{40}$; (c) (d) $BiFeO_3/Bi_2O_3$; (e) (f) $BiFeO_3/Bi_{25}FeO_{40}/Bi_2O_3$; and (g) (h) $Bi_{25}FeO_{40}/Bi_2O_3$. In these figures, C/C_0 represents the concentration changes of MB and $ln(C_0/C)$ represents the reaction rate constant.

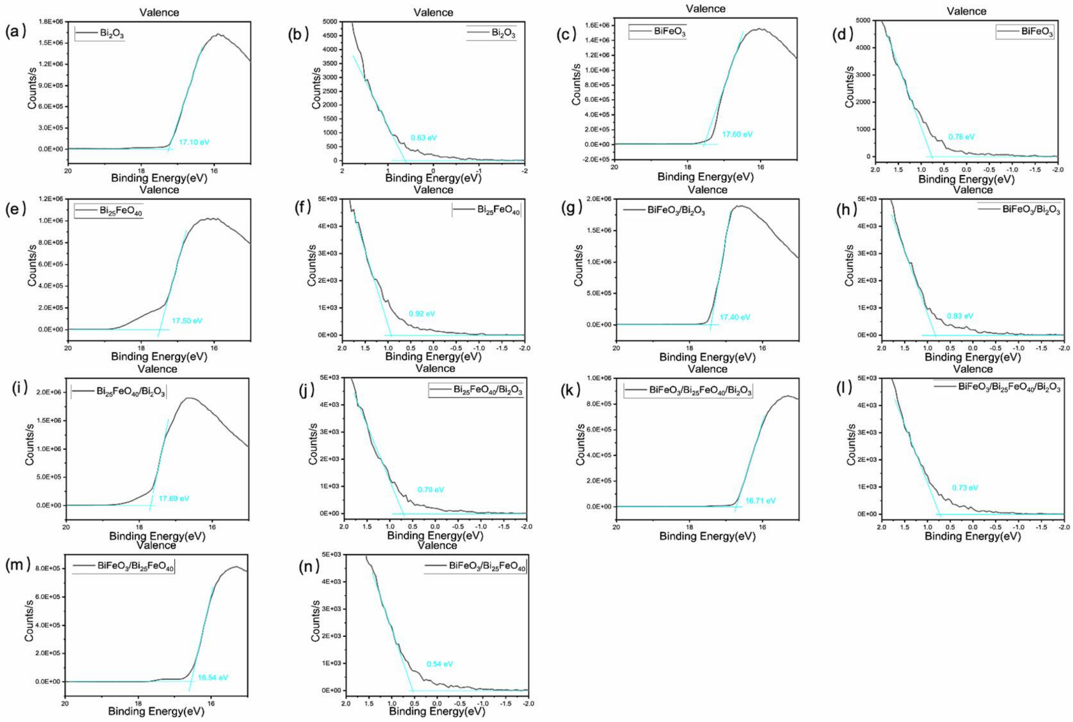

Figure S3. UPS measurement of Bi_2O_3, $BiFeO_3$, $Bi_{25}FeO_{40}$, $BiFeO_3/Bi_2O_3$, $Bi_{25}FeO_{40}/Bi_2O_3$, $BiFeO_3/Bi_{25}FeO_{40}/Bi_2O_3$, and $BiFeO_3/Bi_{25}FeO_{40}$.
